# Sex differences between serum uric acid levels and cardiovascular outcomes in patients with coronary artery disease after stent implantation

**DOI:** 10.3389/fcvm.2023.1021277

**Published:** 2023-02-06

**Authors:** Song Lin Yuan, Moo Hyun Kim, Kwang Min Lee, Xuan Jin, Zhao Yan Song, Jong-Sung Park, Young-Rak Cho, Kyunghee Lim, Sung-Cheol Yun

**Affiliations:** ^1^Department of Cardiology, Dong-A University Hospital, Busan, Republic of Korea; ^2^Department of Clinical Epidemiology and Biostatistics, Asan Medical Center, University of Ulsan College of Medicine, Seoul, Republic of Korea

**Keywords:** sex, serum uric acid, cardiovascular outcomes (CV outcomes), coronary artery disease, stent implantation

## Abstract

**Background:**

The relationship between elevated serum uric acid (SUA) levels and cardiovascular outcomes after stent implantation remains uncertain. This study sought to evaluate the impact of SUA on 12-month cardiovascular outcomes after stent implantation.

**Methods:**

We performed a retrospective study of patients who successfully underwent stent implantation and enrolled 3,222 patients with coronary artery disease (CAD) from a single center. SUA levels were measured before stent implantation. The patients were divided into six groups (<4, 4–4.9, 5–5.9, 6–6.9, 7–7.9 and ≥ 8 mg/dL) at SUA intervals of 1.0 mg/dL. The incidence of cardiovascular outcomes in the six groups was monitored for 1 year after stent implantation and the hazard ratios were estimated. Hazard ratios (HRs) and 95% confidence intervals (95% CIs) for cardiovascular outcomes were estimated using a Cox proportional hazard regression analysis. The primary endpoint was all-cause death. The secondary endpoint was a composite of all-cause death, myocardial infarction, target vessel revascularization, stent thrombosis and stroke. The follow-up duration was 12 months.

**Results:**

Over the 12-month follow-up period, there were 101 all-cause deaths and 218 MACCE. After adjustment for several parameters, the group with SUA levels of more than or equal to 8 mg/dL had significantly higher hazard ratios in the incidence of all-cause death or MACCE. The group with <4.0 mg/dL had significantly higher hazard ratios in all-cause death only in male patients. In contrast, there were no significant differences observed for cardiovascular outcomes in female patients.

**Conclusions:**

Our study identified a U-shaped association between SUA levels and cardiovascular outcomes during 12-month follow-up for males, but not for females. Further studies are warranted to clarify the sex differences between SUA levels and clinical outcomes.

## 1. Introduction

Gertler et al., reported higher levels of serum uric acid (SUA) in patients with early-onset coronary heart disease (CHD) compared to the normal population, and for the first time recommended SUA as a potential risk factor in CHD patients (1). SUA was included in the Framingham Heart study to screen for a potential risk factor for cardiovascular disease (CVD) (2). Thereafter, the relationship between SUA and CVD was extensively investigated, but the nature of the relationship between UA and CVD remains unclear.

Uric acid (UA) is the primary metabolite of purines in the human body and is also a major risk factor for gout, arthritis and kidney stone formation (3). Epidemiological studies have shown that elevated SUA levels are associated with various CVDs (4–6). According to the 2018 Guidelines of Arterial Hypertension, SUA should be assessed to stratify patients for cardiovascular risk factors (7).

In recent years, high as well as low SUA levels have become of increasing concern. Low SUA levels may be harmful to health and lead to undesirable clinical outcomes (8–10). However, only a few studies have examined the impact of SUA levels on cardiovascular outcomes after stent implantation. We undertook the present study to evaluate the relationship between SUA levels and the risk of cardiovascular outcomes after stent implantation.

## 2. Materials and methods

### 2.1. Study design and patient population

Between January 2014 and December 2019, data was collected from patients (*n* = 3,458) who had successfully undergone stent implantation in our single center registry. For this registry, all participants lacking data for SUA levels (*n* = 236) were excluded. Ultimately, 3,222 participants were enrolled in the study including patients with stable angina (*n* = 459, 14.2%), unstable angina (*n* = 1,054, 32.7%), ST-segment elevation myocardial infarction (STEMI, 653, 20.2%), and non-ST-segment myocardial infarction (NSTEMI, 1,056, 32.8%). The patients were divided into six groups (<4, 4–4.9, 5–5.9, 6–6.9, 7–7.9 and ≥ 8 mg/dL) at SUA intervals of 1.0 mg/dL, as follows: Group 1 = those with a SUA level of <4.0 mg/dL; Group 2 = a SUA level between 4.0 and 4.9 mg/dL; Group 3 = a SUA level between 5.0 and 5.9 mg/dL; Group 4 = a SUA level between 6.0 and 6.9 mg/dL; Group 5 = a SUA level between 7.0 and 7.9 mg/dL; Group 6 = a SUA level more than or equal to 8.0 mg/dL. CAD was diagnosed including a history of angina with evidence of ischemic change on electrocardiogram (ECG) recording, and myocardial infarction attack or angina symptoms with significant stenosis lesions in coronary computed tomography angiography (CCTA). Symptomatic patients who received percutaneous coronary intervention (PCI) with stent implantation were eligible for enrolment. The study protocol was conducted in accordance with the Declaration of Helsinki and the ethical standards of the ethics committee of our hospital for human experimentation (DAUHIRB-22-054).

### 2.2. Data collection

The data and study materials were conducted at Dong-A University Hospital in Busan, Republic of Korea. Baseline characteristics were recorded from every patient including age, sex, BMI, ejection fraction (EF), ACS, coronary risk factors (current smoking, hypertension, dyslipidemia and diabetes mellitus), past history (previous MI, previous PCI, previous CABG and previous CVA), number of stents and type of stents. Furthermore, laboratory data assessments (WBC, hemoglobin, platelet, HbA1c, CRP, uric acid, eGFR, AST, ALT, total cholesterol, cTnI) and cardiovascular medications (aspirin, P2Y12 inhibitor, statin, beta blockers, RAS inhibitor, diuretics and gout drugs) were also collected. Of the stent implantation patients, 3,222 had SUA levels measured between admission and stent implantation. If patients had multiple SUA level measurements during the same time period, the one before the time of stent implantation was used for this analysis.

### 2.3. Clinical outcomes and follow-up

The primary endpoint was defined as all-cause death. The secondary endpoint was defined as a composite of all-cause death, myocardial infarction, target vessel revascularization, stent thrombosis and stroke. Follow-up information for all discharged patients was obtained by experienced data technicians at 3, 6, and 12 months after the stent implantation. Patients with cardiac or other chief complaints during follow-up underwent complete clinical evaluation. Follow-up information was collected and adverse events determined by medical staff who were not aware of the uric acid levels.

### 2.4. Statistical analysis

Student's *t*-test, chi-square test and one-way ANOVA were used to compare the baseline characteristics of the study participants between the sexes. The cumulative incidence of cardiovascular outcomes was analyzed using a Kaplan-Meier curve and log-rank test. The reference group was defined as Group 3 in both males and females. HRs with 95% confidence intervals (95% CIs) for cardiovascular outcomes were estimated using Cox proportional hazard regression analysis. Cox proportional hazard models were applied to estimate the HRs of cardiovascular outcomes after adjusting for age, BMI, EF, ACS, CHF, CKD, coronary risk factors, past history, stent type, laboratory assessment (hemoglobin, platelet, HbA_1C_, eGFR and total-cholesterol) and cardiovascular medications. All statistical analyses were conducted using IBM SPSS Version 22 (IBM, Chicago, IL, USA) and R Studio (Integrated Development Environment for R; R Studio, PBC, Boston, MA, USA). Statistical significance was set at *P* < 0.05.

## 3. Results

### 3.1. Baseline clinical characteristics

Patients lacking SUA level (*n* = 236) data were excluded. A total of 3,222 subjects were enrolled in this study. The patients consisted of 2,387 males (74.1%) and females 835 (25.9%). Baseline characteristics of sex are described in Table 1. Compared to male patients, female patients were older; had less frequent hyperuricemia, STEMI, current smoking; had more frequent CKD, hypertension, dyslipidemia, diabetes mellitus; had higher LVEF, platelet, HbA1c, CRP, AST, total cholesterol level; and had lower BMI and SUA levels. They were taking less statin, beta blockers and more diuretics. Baseline characteristics of the study participants by SUA levels by sex are shown in Supplementary Tables 1, 2.

**Table 1:** Baseline characteristics according to sex.

**Variables**	**Overall (*n* = 3,222)**	**Male (*n* = 2,387)**	**Female (*n* = 835)**	***P*-value**
Age, (years)	65.7 ± 11.6	63.4 ± 11.4	72.3 ± 9.4	<0.001
BMI, (kg/m^2^)	24.3 ± 3.1	24.5 ± 3.0	23.9 ± 3.3	<0.001
LVEF, (%)	53.8 ± 9.9	53.6 ± 10.0	54.5 ± 9.9	0.026
Hyperuricemia, *n* (%)	716 (22.2)	585 (24.5)	131 (15.7)	<0.001
ACS, *n* (%)	2,763 (85.8)	2,053 (86.0)	710 (85.0)	0.487
Clinical diagnosis, *n* (%)				0.001
Stable angina	459 (14.2)	334 (14.0)	125 (15.0)	0.487
Unstable angina	1,054 (32.7)	756 (31.7)	298 (35.7)	0.033
STEMI	653 (20.2)	525 (22.0)	128 (15.3)	<0.001
NSTEMI	1,056 (32.8)	772 (32.3)	284 (34.0)	0.376
CHF, *n* (%)	926 (28.7)	692 (29.0)	234 (28.0)	0.592
CKD, *n* (%)	875 (27.2)	532 (22.3)	343 (41.1)	<0.001
**Coronary risk factors**, ***n*** **(%)**				
Current smoking	954 (29.6)	896 (37.5)	58 (6.9)	<0.001
Hypertension	1,798 (55.8)	1,236 (51.8)	562 (67.3)	<0.001
Dyslipidemia	418 (13.0)	277 (11.6)	141 (16.9)	<0.001
Diabetes mellitus	876 (27.2)	554 (23.2)	322 (38.6)	<0.001
**Previous history**, ***n*** **(%)**				
Previous MI	218 (6.8)	156 (6.5)	62 (7.4)	0.378
Previous PCI	378 (11.7)	258 (11.9)	120 (14.4)	0.068
Previous CABG	35 (1.1)	21 (0.9)	14 (1.7)	0.056
Previous CVA	181 (5.6)	124 (5.2)	57 (6.8)	0.078
Number of stents, *n*	1.6 ± 0.8	1.6 ± 0.9	1.6 ± 0.8	0.946
**Types of stents**, ***n*** **(%)**				
DP-DES	1,523 (47.3)	1,110 (46.5)	413 (49.5)	0.140
BP-DES	1,424 (44.2)	1,074 (45.0)	350 (41.9)	0.123
**Laboratory assessment**				
WBC, x10^3^/mm^3^	9.2 ± 3.9	9.3 ± 3.9	9.0 ± 4.0	0.039
Hemoglobin, g/dL	13.4 ± 2.1	13.9 ± 2.0	12.1 ± 1.6	<0.001
Platelet, x10^3^/mm^3^	228.1 ± 69.7	222.4 ± 62.9	244.5 ± 84.3	<0.001
HbA_1C_, %	6.6 ± 1.3	6.6 ± 1.3	6.7 ± 1.3	0.018
CRP, mg/dL	1.4 ± 3.6	1.3 ± 3.5	1.5 ± 3.8	0.124
Uric acid, mg/dL	5.8 ± 1.8	6.0 ± 1.8	5.4 ± 1.9	<0.001
eGFR, mL/min/1.73m^2^	73.4 ± 27.9	76.4 ± 27.6	65.0 ± 27.1	<0.001
AST, U/L	49.3 ± 87.6	48.3 ± 69.4	52.0 ± 126.1	0.422
ALT, U/L	31.5 ± 42.0	32.7 ± 40.2	28.2 ± 46.7	0.008
Total cholesterol, mg/dL	177.0 ± 48.4	175.2 ± 48.6	182.3 ± 47.3	<0.001
cTnI, pg/ml	46.9 ± 1,203.1	59.1 ± 1,394.4	12.1 ± 160.1	0.332
**Medication at discharge**				
Aspirin	3,099 (96.2)	2,302 (96.4)	797 (95.4)	0.180
P2Y12 inhibitor	3,222 (100)	2,387 (100)	835 (100)	-
Statin	2,762 (85.7)	2,066 (86.6)	696 (83.4)	0.021
Beta blockers	2,223 (69.0)	1,683 (70.5)	540 (64.7)	0.002
RAS inhibitor	1,264 (39.2)	919 (38.5)	345 (41.3)	0.154
Diuretics	1,108 (34.4)	722 (30.2)	386 (46.2)	<0.0001
Gout drugs	40 (1.2)	33 (1.4)	7 (0.8)	0.221

Values for categorical and continuous variables are given as numbers (percentages) and means ± standard deviation, respectively.

ACS, acute coronary syndrome; AST, aspartate aminotransaminase; ALT, alanine aminotransaminase; BMI, bodymass index; BP-DES, biodegardable polymer-drug eluting stents; CABG, coronary artery bypass graft; CHF, congestive heart failure; CKD, chronic kidney disease; CRP, C-reactive protein; CVA, cerebrovascular accidents; cTnI, cardiac troponin I; DP-DES, durable polymer-drug eluting stents; eGFR, glomerular filtration rate; HBA1C, hemoglobin A1C; LVEF, left ventricular ejection fraction; MI, myocardial infarction; NSTEM, non-ST-segment myocardial infarction; PCI, percutaneous coronary intervention; RAS, renin-angiotensin system; STEM, ST-elevation myocardial infarction; WBC, white blood cell; -, not available.

### 3.2. Cardiovascular outcomes in male patients

**Figure 1:**
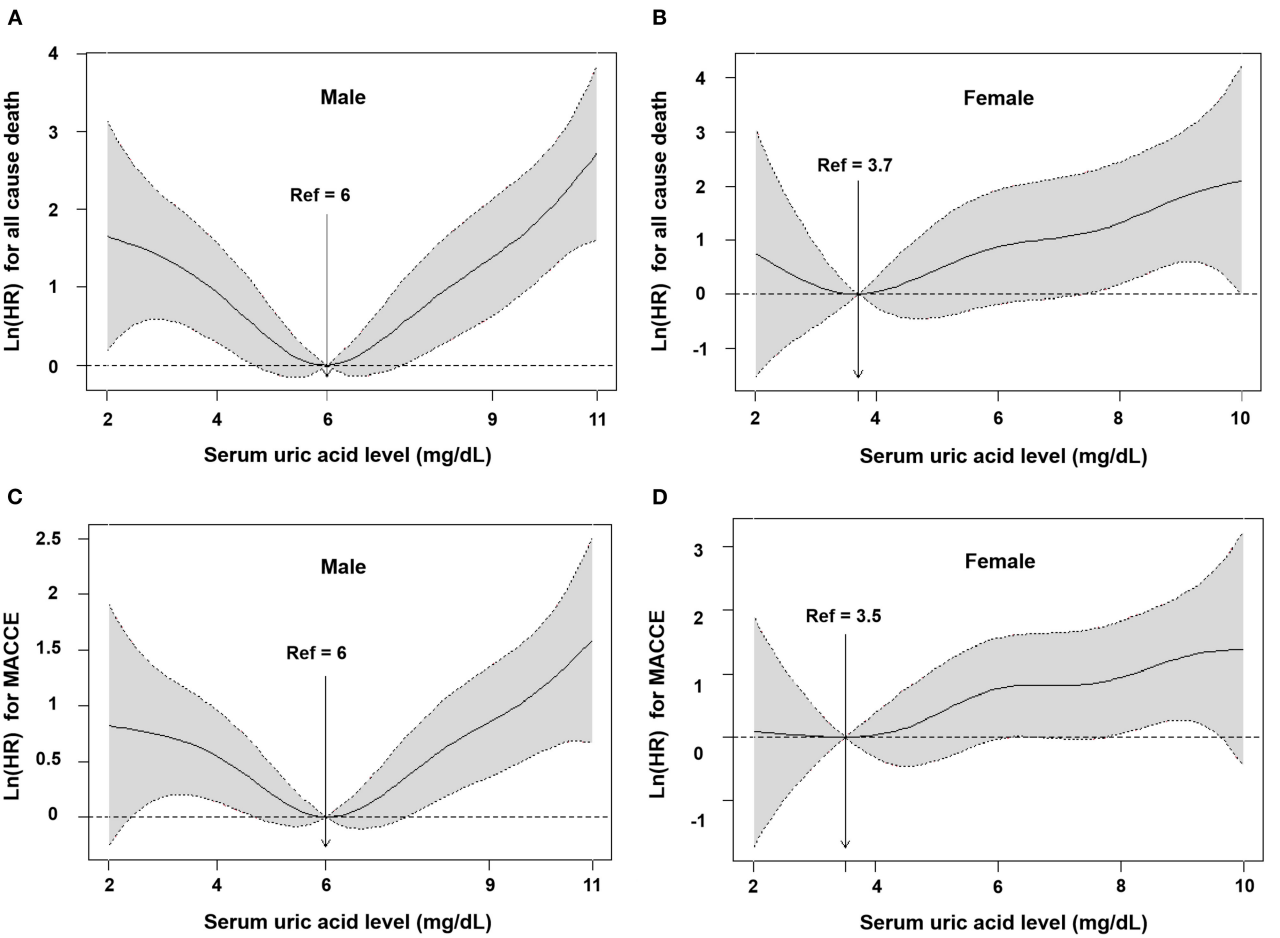
Cubic spline models for association between serum uric acid and outcomes according to sex. All cause death among male **(A)** and female **(B)**, and MACCE among male **(C)** and female **(D)**. Hazard ratio (solid line) from restricted cubic spline modes for all-cause death and MACCE using baseline uric acid levels. Shading represents 95% confidence intervals. HR, hazard ratio; MACCE, major adverse cardiovascular and cerebrovascular events.

In total, 67 deaths and 159 MACCEs occurred within the first year of follow-up. When the subjects were divided into 6 groups (Table 2), in the group with SUA of ≥ 7 mg/dL, higher SUA levels had a higher hazard ratio compared to the group with SUA of 5–5.9 mg/dL (unadjusted HR 3.019, 95% CI 1.097–8.306). Meanwhile, patients who had SUA levels of ≤4.0 mg/dL also had a higher hazard ratio compared with those with SUA of 5–5.9 mg/dL for all-cause death and MACCE (unadjusted HR 4.829, 95% CI 1.813–12.869; unadjusted HR 2.027, 95% CI 1.135–3.621). We added a multivariate analysis to confirm that uric acid was one of the most valuable predictors of cardiovascular events with SUA of 5–5.9 mg/dL. Furthermore, after adjustment for age, BMI, EF, ACS, CHF, CKD, coronary risk factors, stent type, past history and laboratory assessment (hemoglobin, platelet, HbA_1C_, eGFR and total-cholesterol) and cardiovasvular medications, the group with SUA of <4.0 mg/dL and the group with ≥ 8 mg/dL had significantly higher hazard ratios (adjusted HR 2.942, 95% CI 1.057–8.190; adjusted HR 3.897, 95% CI 1.494–10.168) for all-cause death. The group with ≥ 8 mg/dL had significantly higher hazard ratios (HR 2.012, 95% CI 1.180–3.429) in MACCE (Table 2). This U-shaped association between SUA levels and the incidence of all-cause death and MACCE remained consistent after adjustment (Figures 1A, C). Supplementary Figure 1 shows that the higher rate of all-cause death and MACCE was found in the hyperuricemia group (log-rank *p* = 0.006 and log-rank *p* = 0.011).

**Table 2:** Twelve-month all-cause death and MACCE by uric acid levels according to sex.

**Uric acid levels**	**No. of patients/sex**	**No. of patients/events (%)**	**Male**		**Female**	
			**Unadjusted mode HR (95% CI)**	**Adjusted mode HR (95% CI)**	**Unadjusted mode HR (95% CI)**	**Adjusted mode HR (95% CI)**
**All-cause death**						
<4 mg/dL	235/184	12 (5.1)/5 (2.7)	4.829 (1.813–12.868)^**^	2.942 (1.057–8.190)^*^	0.696 (0.228–2.128)	2.100 (0.578–7.628)
4–4.9 mg/dL	454/188	14 (3.1)/4 (2.1)	2.889 (1.110–7.517)^*^	3.193 (1.183–8.618)^*^	0.547 (0.165–1.816)	0.885 (0.231–3.396)
5–5.9 mg/dL	555/207	6 (1.1)/8 (3.9)	1 (reference)	1 (reference)	1 (reference)	1 (reference)
6–6.9 mg/dL	525/116	7 (1.3)/6 (5.2)	1.233 (0.414–3.668)	1.259 (0.414–3.830)	1.348 (0.468–3.886)	2.379 (0.741–7.637)
7–7.9 mg/dL	309/60	10 (3.2)/3 (5.0)	3.019 (1.097–8.306)^*^	2.865 (1.003 ~ 8.182)^*^	1.316 (0.349–4.959)	1.872 (0.427–8.213)
≥ 8 mg/dL	309/80	18 (5.8)/8 (10.0)	5.536 (2.198–13.947)^***^	3.897 (1.494–10.168)^**^	2.658 (0.997–7.082)	1.275 (0.428–3.800)
***P**-* **value**			0.001	0.029	0.095	0.628
**MACCE**						
<4 mg/dL	235/184	21 (8.9)/10 (5.4)	2.027 (1.135–3.621)^*^	1.678 (0.927–3.037)	0.697 (0.316–1.536)	0.909 (0.395–2.095)
4–4.9 mg/dL	454/188	33 (7.3)/7 (3.7)	1.629 (0.969–2.740)	1.619 (0.955–2.745)	0.473 (0.194–1.149)	0.635 (0.254–1.591)
5–5.9 mg/dL	555/207	25 (4.5)/16 (7.7)	1 (reference)	1 (reference)	1 (reference)	1 (reference)
6–6.9 mg/dL	525/116	24 (4.6)/11 (9.5)	1.012 (0.578–1.772)	0.974 (0.553–1.713)	1.253 (0.581–2.699)	1.156 (0.521–2.562)
7–7.9 mg/dL	309/60	21 (6.8)/5 (8.3)	1.522 (0.852–2.718)	1.389 (0.769–2.509)	1.088 (0.399–2.969)	1.081 (0.373–3.132)
≥ 8 mg/dL	309/80	35 (11.3)/10 (12.5)	2.623 (1.570–4.383)^***^	2.012 (1.180–3.429)^*^	1.645 (0.747–3.625)	0.951 (0.397–2.279)
***P**-* **value**			0.001	0.042	0.141	0.914

CI indicates confidence interval; HR, hazard ratio; MACCE, major adverse cardiovascular and cerebrovascular events. Unadjusted mode included 6 group uric acid levels. Adjusted mode included age, BMI, EF, ACS, CHF, CKD, coronary risk factors, stent type, past history, laboratory assessment (hemoglobin, platelet, HbA1C, eGFR and total-cholesterol) and medication. ^*^p < 0.05, ^**^p < 0.01, ^***^p < 0.001.

### 3.3. Cardiovascular outcomes in female patients

In total, 34 deaths and 59 MACCEs occurred within the first year of follow-up. When the subjects were divided into 6 groups (Table 2), there was no significant difference between the 6 groups (*p* = 0.095 and *p* = 0.141). After adjustment for age, BMI, EF, ACS, CHF, CKD, coronary risk factors, stent type, past history, laboratory assessment (hemoglobin, platelet, HbA_1C_, eGFR and total-cholesterol) and cardiovasvular medications, there was no significant difference between the 6 groups (*p* = 0.628 and *p* = 0.914). The group with SUA of <4.0 mg/dL and the groups with ≥ 8 mg/dL did not have significantly higher hazard ratios for all-cause death and MACCE (Table 2). The U-shape was not existed with the incidence of all-cause death and MACCE after adjustment (Figures 1B, D). Supplementary Figure 1 shows that the highest rate of all-cause death was found in the hyperuricemia group (Log rank *p* = 0.006), but there was no significant difference between the hyperuricemia group and normouricemia group in MACCE (Log rank *p* = 0.077).

### 3.4. Subgroup analysis of ACS and CCS patients according to SUA levels

There was no significant differences between ACS and CCS, and U-shape pattern was maintained in male patients (Supplementary Figures 2A, C), but not in female patients (Supplementary Figures 2B, D).

## 4. Discussion

Our analysis revealed a significant U-shaped association between SUA levels and cardiovascular outcomes after stent implantation in male patients. Interestingly, no such association between baseline SUA levels and cardiovascular outcomes was observed in female patients.

Hyperuricemia is an epidemiological problem, especially if present in association with cardiovascular comorbidities (11). Among patients with acute coronary syndrome (ACS) and chronic coronary syndrome (CCS), patients with hyperuricemia increase significantly to 23% (12–14). The proportion of patients with hyperuricemia in our study (22.2%) was similar to them. The association between increased risk of cardiovascular outcomes with SUA levels in general populations or high-risk cardiovascular disease has been reported. Most studies had focused on the effect of hyperuricemia on clinical outcomes after PCI (15–18). However, Several studies had described outcomes related to U-shaped association between cardiovascular outcomes and SUA level after PCI (19, 20). Guidelines from the American College of Rheumatology for the Management of Gout recommend that symptomatic hyperuricemia be defined at SUA levels of ≥ 6.8 mg/dl with no prior gout flares or subcutaneous tophi (21). According to the Japanese Society of Gout and Nucleic Acid Metabolism, hyperuricemia is defined as SUA levels of >7.0 mg/dL, regardless of sex (22). Previous studies showed that SUA levels of >6.8 mg/dl are associated with higher mortality in myocardial infarction (16, 23). However, those levels do not represent a true cut-off value for preventing cardiovascular diseases (they are relevant for gout prevention). Furthermore, the SUA threshold for preventing cardiovascular diseases has not been established to date. Hyperuricemia is a risk factor for cardiovascular and kidney diseases, but SUA levels from which this risk begins to increase remains unknown and could be lower than 6 mg/dl (24).

U or J-shaped associations between SUA levels and the incidence of cardiovascular events have been observed in previous studies (25–30), although the mechanism responsible has not been identified. Our study highlights the necessity of addressing the risk of non-hyperuricemia in addition to hyperuricemia in the pathogenesis of cardiovascular disease. The causal relationship between uric acid and cardiovascular events remains uncertain and determining the optimal range of uric acid control in patients with non-hyperuricemia is challenging. Furthermore, the strength of the relationship between uric acid and cardiovascular events suggests that uric acid may be used for risk stratification in patients with CAD. Ichiro et al., reported that 1,949 patients underwent successful PCI and the results showed a J-shaped relationship between SUA levels and cardiovascular events, although sex differences were not evaluated (19). A Korean cohort study demonstrated that low SUA levels are independently associated with increased risk of all-cause mortality in males and females, an increased risk of CVD mortality in females, and an increased risk of cancer mortality in males (25). This study included young and middle-aged Korean males and females who regularly attended health screening examinations. Another Korean cohort study of 27,490 participants with normal kidney function demonstrated that low SUA levels (≤4 mg/dL) were an independent risk factor for all-cause mortality in males, while high SUA levels (> 8 mg/dL) were not. There was no significant relationship between SUA levels and mortality in females (26). This data is similar to our study. A longitudinal Taiwanese study of elderly health screening examination program in older people (>65 years old) showed that SUA levels ≥ 8 or <4 mg/dL were indicative of higher mortality risk and malnourishment (excluding hospitalized older people), regardless of sex (27). In our findings, an association between low and high uric acid levels (<4 mg/dL and ≥8 mg/dL) and all-cause death and MACCE was identified in male patients, but not restricted by age over 65 or with consideration of nutritional status. Cohorts in a Japanese study of 36,313 subjects showed J- or U-shaped relationships between serum uric acid levels and cardiovascular mortality, but did not consider the impact of HbA1C, and eGFR, etc. (28). Kuo et al., analyzed 354,110 subjects without a history of gout and suggested SUA levels of 0.30–0.41 mmol/L (5.04–6.89 mg/dL) were associated with the lowest incidence of all cause and cardiovascular mortality and should be regarded as optimal (29). However, that study did not include smoking status, alcohol intake or BMI as covariates. Recently, a Chinese cohort study of 3,047 participants excluding severe heart failure and eGFR <30 min per 1.73 m^2^ showed a U-shaped relationship with all cause death and cardiovascular outcomes in high atherosclerotic risk patients, regardless of sex (30). A previous study showed that SUA levels in adult males are on average ~1 mg/dl higher than in females, but SUA levels in females increased significantly around natural menopause (31). With menopause, lower estrogen levels and increased SUA levels may promote the development of endothelial dysfunction and CVD (32, 33). Our study showed a U-shaped association between SUA levels and cardiovascular outcomes in male patients. However, female patients only numerically showed a J-shaped correlation trend in cardiovascular outcomes. This is partly due to the low number of female patients in our study, who were also older and had lower uric acid levels compare to male patients (5.4 vs. 6.0 mg/dL, *p* = 0.001, Table 1). This trend is more evident if the low reference (4.0–4.9 mg/dL) was used (Supplementary Table 3).

Several potential mechanisms have been proposed to explain why elevated uric acid levels may lead to cardiovascular outcomes. Direct endothelial injury, increasing oxidative stress, decreased NO (nitric oxide) and systemic inflammatory response underlies the negative mechanisms (34). The production of oxygen free radicals has been related to the catabolism of xanthine oxidase (XO), which can damage endothelial cells, vascular smooth muscle cells, and atrial muscle cells, etc. (35). As the concentration of uric acid increases, it can gradually penetrate directly vascular endothelial cells *via* urate transporters (GLUT9/URATv1), leading to endothelial dysfunction and proliferation of vascular smooth muscle cells (36), which is also a manifestation of active inflammation (37). In patients with CAD, XO activity is increased and NO activation is reduced, while elevated uric acid is closely associated with visceral fat accumulation and insulin resistance. Uric acid is an important antioxidant and free radical scavenger *in vitro*, thus hypouricemia increases risk of atherosclerotic diseases owing to decreased antioxidant capacity (38). Low SUA levels may indicate a decrease in total antioxidant capacity and malnourishment (27).

## 5. Limitations

There are some limitations to our study that warrant consideration. This was a single-center, retrospective study and we only recorded 1 year of follow up for cardiovascular outcomes and did not distinguish between cardiac and non-cardiac deaths, due to the relatively small number of overall patients. Therefore, the occurrence of cardiovascular outcomes may be affected by the stratification of SUA levels. Meanwhile, compared with the large sample data (25–28), the clinical outcomes (U-shaped curve) in our study exhibited a significant right shift. Second, a single assessment of SUA levels at baseline was used to predict cardiovascular outcomes, rather than variability of SUA levels. Third, our study did not take into account information on risk factors for chronic hyperuricaemia (dietary intake, nutrition status, etc). Fourth, this study's findings are subject to the well-known limitations of a retrospective design. The associations between high-sensitivity C-reactive protein, B-type natriuretic peptide, and other proinflammatory cytokines and markers of oxidative stress were not evaluated as part of a multivariable model. Fifth, there is a risk of bias from these data which may be explored, but unlikely to be resolved by sensitivity analysis, therefore leaving a consistent risk of spuriousness. Therefore, attention should be paid to interpreting the results of this study.

## 6. Conclusions

Our study identified a U-shaped association between SUA levels and cardiovascular outcomes during 12-month follow-up for males, but not for females. Further studies are warranted to clarify the sex differences between SUA levels and clinical outcomes.

## Data availability statement

The raw data supporting the conclusions of this article will be made available by the authors, without undue reservation.

## Author contributions

MHK and SY contributed to the study concept and design, contributed to drafting for the manuscript, and critical revision of the manuscript for important intellectual content. SY, XJ, and ZS acquired the data. MHK supervised the progress of the study. SY, KML, and S-CY contributed to the acquisition, formal analysis, and interpretation of data. J-SP, Y-RC, and KL contributed to validation and correction. All authors listed have made a substantial, direct, and intellectual contribution to the work and approved it for publication.
