# Supplementary material for: Sex differences between serum uric acid levels and cardiovascular outcomes in patients with coronary artery disease after stent implantation
Source: Front Cardiovasc Med. 2023 Feb 6;10:1021277. doi: 10.3389/fcvm.2023.1021277 (PMC9939523; doi:10.3389/fcvm.2023.1021277)
Supplement: Supplementary file 1 [file Data_Sheet_1.docx]

**Supplementary Material**

**Table 1S. Comparison of subjects’ baseline characteristics of male according to serum uric acid levels.**

| **Variables** | **Male** | | | | | | ***P-*value** |
| --- | --- | --- | --- | --- | --- | --- | --- |
|  | **SUA levels, mg/dL** | | | | | |  |
|  | **< 4** | **4 - 4.9** | **5 - 5.9** | **6 - 6.9** | **7 - 7.9** | **≥ 8** |  |
| Number | 235 | 454 | 555 | 525 | 309 | 309 |  |
| Age, (years) | 65.7 ± 10.2 | 63.9 ± 10.9 | 62.9 ± 11.6 | 62.2 ± 10.7 | 63.4 ± 11.9 | 64.3 ± 13.1 | 0.002 |
| BMI, (kg/m^2^) | 23.7 ± 3.1 | 24.1 ± 2.9 | 24.3 ± 2.9 | 24.8 ± 3.0 | 24.9 ± 3.0 | 24.8 ± 3.4 | <0.001 |
| LVEF, (%) | 53.9 ± 9.4 | 53.8 ± 9.9 | 54.2 ± 9.7 | 53.9 ± 9.8 | 53.0 ± 10.3 | 51.9 ± 10.6 | 0.026 |
| ACS, n (%) | 191 (81.3) | 385 (84.8) | 489 (88.1) | 455 (86.7) | 270 (87.4) | 263 (85.1) | 0.166 |
| **Clinical diagnosis, n (%)** |  |  |  |  |  |  | 0.004 |
| Stable angina | 44 (18.7) | 69 (15.2) | 66 (11.9) | 70 (13.3) | 39 (12.6) | 46 (14.9) | 0.166 |
| Unstable angina | 88 (37.4) | 152 (33.5) | 191 (34.4) | 161 (30.7) | 94 (30.4) | 70 (22.7) | 0.003 |
| STEMI | 35 (14.9) | 95 (20.9) | 122 (22.0) | 111 (21.1) | 77 (24.9) | 85 (27.5) | 0.013 |
| NSTEMI | 68 (28.9) | 138 (30.4) | 176 (31.7) | 183 (34.9) | 99 (32.0) | 108 (35.0) | 0.463 |
| CHF, n (%) | 65 (27.7) | 127 (28.0) | 155 (27.9) | 148 (28.2) | 99 (32.0) | 98 (31.7) | 0.637 |
| CKD, n (%) | 39 (16.6) | 45 (9.9) | 87 (15.7) | 99 (18.9) | 89 (28.8) | 173 (56.0) | <0.001 |
| **Coronary risk factors, n (%)** |  |  |  |  |  |  |  |
| Current smoking | 70 (29.8) | 168 (37.0) | 232 (41.8) | 213 (40.6) | 114 (36.9) | 99 (32.0) | 0.013 |
| Hypertension | 124 (52.8) | 200 (44.1) | 265 (47.7) | 290 (55.2) | 164 (53.1) | 193 (62.5) | <0.001 |
| Dyslipidemia | 26 (11.1) | 58 (12.8) | 54 (9.7) | 67 (12.8) | 40 (12.9) | 32 (10.4) | 0.511 |
| Diabetes mellitus | 67 (28.5) | 118 (26.0) | 113 (20.4) | 117 (22.3) | 60 (19.4) | 79 (25.6) | 0.036 |
| **Previous history, n (%)** |  |  |  |  |  |  |  |
| Previous MI, n (%) | 25 (10.6) | 31 (6.8) | 23 (4.1) | 32 (6.1) | 24 (7.8) | 21 (6.8) | 0.026 |
| Previous PCI, n (%) | 33 (14.0) | 72 (15.9) | 53 (9.5) | 59 (11.2) | 27 (8.7) | 41 (13.3) | 0.013 |
| Previous CABG, n (%) | 2 (0.9) | 7 (1.5) | 2 (0.4) | 2 (0.4) | 2 (0.6) | 6 (1.9) | 0.085 |
| Previous CVA, n (%) | 20 (8.5) | 19 (4.2) | 24 (4.3) | 21 (4.0) | 13 (4.2) | 27 (8.7) | 0.004 |
| Number of stents, n | 1.6 ± 1.0 | 1.5 ± 0.8 | 1.5 ± 0.8 | 1.6 ± 0.9 | 1.5 ± 0.8 | 1.6 ± 0.9 | 0.611 |
| Types of stents, n (%) |  |  |  |  |  |  |  |
| DP-DES | 109 (46.4) | 211 (46.5) | 275 (49.5) | 242 (46.1) | 135 (43.7) | 138 (44.7) | 0.622 |
| BP-DES | 106 (45.1) | 185 (40.7) | 251 (45.2) | 239 (45.5) | 147 (47.6) | 146 (47.2) | 0.435 |
| **Laboratory assessment** |  |  |  |  |  |  |  |
| WBC, x10^3^/mm^3^ | 9.1 ± 3.7 | 9.1 ± 3.9 | 9.2 ± 3.6 | 9.2 ± 3.6 | 9.1 ± 3.8 | 10.5 ± 4.9 | <0.001 |
| Hemoglobin, g/dL | 13.4 ± 2.2 | 13.9 ± 1.8 | 14.0 ± 1.9 | 14.1 ± 2.0 | 13.9 ± 2.0 | 13.5 ± 2.5 | <0.001 |
| Platelet, x10^3^/mm^3^ | 226.0 ± 66.6 | 219.2 ± 57.3 | 228.1 ± 67.3 | 222.8 ± 59.9 | 216.3 ± 58.5 | 219.4 ± 67.8 | 0.070 |
| HbA_1C_, % | 6.9 ± 1.5 | 6.7 ± 1.4 | 6.5 ± 1.2 | 6.6 ± 1.3 | 6.4 ± 1.1 | 6.5 ± 1.0 | <0.001 |
| CRP, mg/dL | 2.0 ± 4.3 | 1.1 ± 2.9 | 1.2 ± 3.2 | 1.0 ± 3.0 | 1.3 ± 3.8 | 1.9 ± 4.3 | <0.001 |
| Uric acid, mg/dL | 3.2 ± 0.7 | 4.5 ± 0.3 | 5.4 ± 0.3 | 6.4 ± 0.3 | 7.4 ± 0.3 | 9.1 ± 1.1 | <0.001 |
| eGFR, mL/min/1.73m^2^ | 84.9 ± 41.8 | 85.7 ± 22.5 | 80.2 ± 23.4 | 75.6 ± 21.6 | 69.3 ± 25.9 | 57.6 ±27.6 | <0.001 |
| AST, U/L | 41.6 ± 43.5 | 48.5 ± 53.2 | 43.0 ± 50.4 | 49.0 ± 92.6 | 45.9 ± 65.1 | 63.9 ± 89.2 | 0.001 |
| ALT, U/L | 28.1 ± 27.6 | 28.5 ± 19.0 | 31.7 ± 42.1 | 34.1 ± 52.2 | 31.8 ± 26.9 | 42.6 ± 52.3 | <0.001 |
| Total cholesterol, mg/dL | 161.1 ± 42.9 | 168.7 ± 46.8 | 177.6 ± 47.0 | 180.3 ± 46.4 | 180.1 ± 57.7 | 177.5 ± 49.4 | <0.001 |
| cTnI, pg/ml | 20.5 ± 239.5 | 4.2 ± 14.2 | 90.6 ± 2063.7 | 14.2 ± 258.4 | 130.7 ± 2189.8 | 117.1 ± 1558.7 | 0.691 |
| **Medication at discharge** |  |  |  |  |  |  |  |
| Aspirin | 220 (93.6) | 446 (98.2) | 531 (95.7) | 510 (97.1) | 300 (97.1) | 295 (95.5) | 0.027 |
| P2Y12 inhibitor | 235 (100.0) | 454 (100.0) | 555 (100.0) | 525 (100.0) | 309 (100.0) | 309 (100.0) | - |
| Statin | 189 (80.4) | 411 (90.5) | 491 (88.5) | 467 (89.0) | 265 (85.8) | 243 (78.6) | <0.001 |
| Beta blockers | 154 (65.5) | 315 (69.4) | 398 (71.7) | 373 (71.0) | 227 (73.5) | 216 (69.9) | 0.430 |
| RAS inhibitor | 84 (35.7) | 166 (36.6) | 221 (39.8) | 208 (39.6) | 117 (37.9) | 123 (39.8) | 0.786 |
| Diuretics | 60 (25.5) | 134 (29.5) | 135 (24.3) | 150 (28.6) | 95 (30.7) | 148 (47.9) | <0.001 |
| Gout drugs | 9 (3.8) | 4 (0.9) | 3 (0.5) | 6 (1.1) | 2 (0.6) | 9 (2.9) | 0.001 |
| Values for categorical and continuous variables are given as numbers (percentages) and means ± standard deviation, respectively. ACS, acute coronary syndrome. AST, aspartate aminotransaminase. ALT, alanine aminotransaminase. BMI, body mass index. BP-DES, biodegardable polymer-drug eluting stents. CABG, coronary artery bypass graft. CHF, congestive heart failure. CKD, chronic kidney disease. CRP, C-reactive protein. CVA, cerebrovascular accidents. cTnI= cardiac troponin I. DP-DES, durable polymer-drug eluting stents. eGFR, glomerular filtration rate. HBA1C, hemoglobin A1C. LVEF, left ventricular ejection fraction. MI, myocardial infarction, NSTEM, non-ST-segment myocardial infarction. PCI, percutaneous coronary intervention. RAS, renin-angiotensin system. STEM, ST-elevation myocardial infarction. WBC, white blood cell. -, not available. | | | | | | | |

**Table 2S. Comparison of subjects’ baseline characteristics of female according to serum uric acid levels.**

| **Variables** | **Female** | | | | | | ***P-*value** |
| --- | --- | --- | --- | --- | --- | --- | --- |
|  | **SUA levels, mg/dL** | | | | | |  |
|  | **< 4** | **4 - 4.9** | **5 - 5.9** | **6 - 6.9** | **7 - 7.9** | **≥ 8** |  |
| Number | 184 | 188 | 207 | 116 | 60 | 80 |  |
| Age, (years) | 71.4 ± 9.2 | 70.0 ± 10.1 | 72.3 ± 9.0 | 72.9 ± 8.8 | 76.0 ± 8.1 | 76.1 ± 9.5 | <0.001 |
| BMI, (kg/m^2^) | 23.5 ± 3.3 | 24.3 ± 3.1 | 24.1 ± 3.2 | 23.6 ± 3.4 | 24.2 ± 2.9 | 23.8 ± 4.2 | 0.309 |
| LVEF, (%) | 56.0 ± 9.0 | 55.9 ± 8.4 | 54.4 ± 10.6 | 53.2 ± 11.5 | 53.2 ± 10.2 | 50.7 ± 9.5 | <0.001 |
| ACS, n (%) | 163 (88.6) | 164 (87.2) | 176 (85.0) | 92 (79.3) | 54 (90.0) | 61 (76.3) | 0.042 |
| **Clinical diagnosis, n (%)** |  |  |  |  |  |  | 0.004 |
| Stable angina | 21 (11.4) | 24 (12.8) | 31 (15.0) | 24 (20.7) | 6 (10.0) | 19 (23.8) | 0.042 |
| Unstable angina | 78 (42.4) | 75 (39.9) | 74 (35.7) | 41 (35.3) | 19 (31.7) | 11 (13.8) | <0.001 |
| STEMI | 30 (16.3) | 31 (16.5) | 26 (12.6) | 17 (14.7) | 8 (13.3) | 16 (20.0) | 0.681 |
| NSTEMI | 55 (29.9) | 58 (30.9) | 76 (36.7) | 34 (29.3) | 27 (45.0) | 34 (42.5) | 0.080 |
| CHF, n (%) | 44 (23.9) | 43 (22.9) | 60 (29.0) | 35 (30.2) | 20 (33.3) | 32 (40.0) | 0.053 |
| CKD, n (%) | 36 (19.6) | 34 (18.1) | 85 (41.1) | 70 (60.3) | 42 (70.0) | 76 (95.0) | <0.001 |
| **Coronary risk factors, n (%)** |  |  |  |  |  |  |  |
| Current smoking | 7 (3.8) | 19 (10.1) | 12 (5.8) | 11 (9.5) | 3 (5.0) | 6 (7.5) | 0.281 |
| Hypertension | 107 (58.2) | 121 (64.4) | 137 (66.2) | 85 (73.3) | 49 (81.7) | 63 (78.8) | 0.001 |
| Dyslipidemia | 26 (14.1) | 45 (23.9) | 30 (14.5) | 18 (15.5) | 10 (16.7) | 12 (15.0) | 0.115 |
| Diabetes mellitus | 68 (37.0) | 68 (36.2) | 80 (38.6) | 36 (31.0) | 27 (45.0) | 43 (53.8) | 0.031 |
| **Previous history, n (%)** |  |  |  |  |  |  |  |
| Previous MI, n (%) | 13 (7.1) | 11 (5.9) | 14 (6.8) | 12 (10.3) | 7 (11.7) | 5 (6.3) | 0.547 |
| Previous PCI, n (%) | 28 (15.2) | 23 (12.2) | 27 (13.0) | 24 (20.7) | 11 (18.3) | 7 (8.8) | 0.175 |
| Previous CABG, n (%) | 2 (1.1) | 4 (2.1) | 3 (1.4) | 4 (3.4) | 1 (1.7) | 0 (0.0) | 0.513 |
| Previous CVA, n (%) | 13 (7.1) | 16 (8.5) | 13 (6.3) | 8 (6.9) | 2 (3.3) | 5 (6.3) | 0.829 |
| Number of stents, n | 1.5 ± 0.7 | 1.5 ± 0.7 | 1.6 ± 0.9 | 1.5 ± 0.9 | 1.6 ± 0.8 | 1.7 ± 0.8 | 0.412 |
| Types of stents, n (%) |  |  |  |  |  |  |  |
| DP-DES | 95 (51.6) | 84 (44.7) | 104 (50.2) | 60 (51.7) | 27 (45.0) | 43 (53.8) | 0.636 |
| BP-DES | 74 (40.2) | 85 (45.2) | 83 (40.1) | 51 (44.0) | 27 (45.0) | 30 (37.5) | 0.789 |
| **Laboratory assessment** |  |  |  |  |  |  |  |
| WBC, x10^3^/mm^3^ | 8.8 ± 5.1 | 8.8 ± 3.1 | 8.3 ± 3.0 | 9.4 ± 3.8 | 9.1 ± 3.3 | 10.8 ± 5.0 | <0.001 |
| Hemoglobin, g/dL | 12.3 ± 1.5 | 12.5 ± 1.3 | 12.1 ± 1.5 | 12.0 ± 2.1 | 11.3 ± 1.5 | 11.1 ± 1.8 | <0.001 |
| Platelet, x10^3^/mm^3^ | 246.7 ± 111.7 | 243.9 ± 68.0 | 243.8 ± 73.9 | 240.7 ± 78.5 | 246.4 ± 81.8 | 246.2 ± 82.9 | 0.994 |
| HbA_1C_, % | 6.6 ± 1.1 | 6.7 ± 1.3 | 6.8 ± 1.5 | 6.7 ± 1.2 | 6.6 ± 1.0 | 6.9 ± 1.2 | 0.371 |
| CRP, mg/dL | 1.0 ± 2.3 | 1.2 ± 4.0 | 0.9 ± 2.2 | 1.9 ± 4.0 | 1.9 ± 3.0 | 4.4 ± 7.1 | <0.001 |
| Uric acid, mg/dL | 3.2 ± 0.6 | 4.5 ± 0.3 | 5.5 ± 0.3 | 6.4 ± 0.3 | 7.4 ± 0.3 | 9.2 ± 1.6 | <0.001 |
| eGFR, mL/min/1.73m^2^ | 80.4 ± 26.5 | 76.0 ± 22.4 | 64.0 ± 22.0 | 55.7 ± 23.2 | 47.0 ± 21.2 | 32.6 ± 16.3 | <0.001 |
| AST, U/L | 46.2 ± 46.9 | 38.9 ± 32.5 | 44.6 ± 60.0 | 53.5 ± 77.0 | 47.9 ± 56.9 | 116.3 ± 367.3 | <0.001 |
| ALT, U/L | 24.3 ± 18.7 | 21.5 ± 12.3 | 26.4 ± 35.2 | 28.1 ± 26.6 | 25.2 ± 21.5 | 59.8 ± 126.5 | <0.001 |
| Total cholesterol, mg/dL | 175.9 ± 44.8 | 183.6 ± 46.6 | 187.1 ± 48.3 | 179.9 ± 46.6 | 188.8 ± 46.4 | 179.8 ± 52.4 | 0.199 |
| cTnI, pg/ml | 3.3 ± 9.3 | 9.4 ± 92.9 | 24.1 ± 300.0 | 16.3 ± 98.7 | 4.1 ± 14.5 | 7.5 ± 18.3 | 0.840 |
| **Medication at discharge** |  |  |  |  |  |  |  |
| Aspirin | 178 (96.7) | 182 (96.8) | 198 (95.7) | 113 (97.4) | 56 (93.3) | 70 (87.5) | 0.011 |
| P2Y12 inhibitor | 184 (100.0) | 188 (100.0) | 207 (100.0) | 116 (100.0) | 60 (100.0) | 80 (100.0) | - |
| Statin | 159 (86.4) | 159 (84.6) | 178 (86.0) | 91 (78.4) | 51 (85.0) | 58 (72.5) | 0.044 |
| Beta blockers | 119 (64.7) | 122 (64.9) | 135 (65.2) | 73 (62.9) | 39 (65.0) | 52 (65.0) | 0.999 |
| RAS inhibitor | 79 (42.9) | 80 (42.6) | 85 (41.1) | 48 (41.4) | 25 (41.7) | 28 (35.0) | 0.896 |
| Diuretics | 64 (34.8) | 62 (33.0) | 97 (46.9) | 67 (57.8) | 37 (61.7) | 59 (73.8) | <0.001 |
| Gout drugs | 3 (1.6) | 0 (0.0) | 0 (0.0) | 2 (1.7) | 1 (1.7) | 1 (1.3) | 0.262 |
| Values for categorical and continuous variables are given as numbers (percentages) and means ± standard deviation, respectively. ACS, acute coronary syndrome. AST, aspartate aminotransaminase. ALT, alanine aminotransaminase. BMI, body mass index. BP-DES, biodegardable polymer-drug eluting stents. CABG, coronary artery bypass graft. CHF, congestive heart failure. CKD, chronic kidney disease. CRP, C-reactive protein. CVA, cerebrovascular accidents. cTnI= cardiac troponin I. DP-DES, durable polymer-drug eluting stents. eGFR, glomerular filtration rate. HBA1C, hemoglobin A1C. LVEF, left ventricular ejection fraction. MI, myocardial infarction, NSTEM, non-ST-segment myocardial infarction. PCI, percutaneous coronary intervention. RAS, renin-angiotensin system. STEM, ST-elevation myocardial infarction. WBC, white blood cell. -, not available. | | | | | | | |

**Table 3S. 12-month all-cause death and MACCE by uric acid levels according to female**

| **Uric acid levels** | **No. of patients** | **Events (%)** | **Female** | |
| --- | --- | --- | --- | --- |
|  |  |  | **Unadjusted mode HR (95% CI)** | **Adjusted mode HR (95% CI)** |
| **All-cause death** |  |  |  |  |
| < 3 mg/dL | 53 | 4 (7.5) | 3.610 (0.903 - 14.435) | 8.249 (1.623 - 41.918)* |
| 3 - 3.9 mg/dL | 131 | 1 (0.8) | 0.354 (0.040 - 3.171) | 0.342 (0.037 - 3.184) |
| 4 - 4.9 mg/dL | 188 | 4 (2.1) | **1 (reference)** | **1 (reference)** |
| 5 - 5.9 mg/dL | 207 | 8 (3.9) | 1.828 (0.551 - 6.071) | 0.774 (0.210 - 2.856) |
| 6 - 6.9 mg/dL | 116 | 6 (5.2) | 2.465 (0.696 - 8.736) | 1.174 (0.290 - 4.748) |
| ≥ 7 mg/dL | 140 | 11 (7.9) | 3.801 (1.210 - 11.938)* | 1.127 (0.300 - 4.225) |
| ***P****-***value** |  |  | 0.067 | 0.055 |
| **MACCE** |  |  |  |  |
| < 3 mg/dL | 53 | 5 (9.4) | 2.641 (0.838 - 8.322) | 2.866 (0.859 - 9.556) |
| 3 - 3.9 mg/dL | 131 | 5 (3.8) | 1.023 (0.325 - 3.233) | 0.918 (0.286 - 2.949) |
| 4 - 4.9 mg/dL | 188 | 7 (3.7) | **1 (reference)** | **1 (reference)** |
| 5 - 5.9 mg/dL | 207 | 16 (7.7) | 2.116 (0.870 - 5.143) | 1.478 (0.590 - 3.706) |
| 6 - 6.9 mg/dL | 116 | 11 (9.5) | 2.650 (1.027 - 6.836)* | 1.529 (0.555 - 4.215) |
| ≥ 7 mg/dL | 140 | 15 (10.7) | 2.973 (1.212 - 7.291)* | 1.426 (0.513 - 3.964) |
| ***P****-***value** |  |  | 0.105 | 0.549 |
| CI indicates confidence interval; HR, hazard ratio; MACCE=major adverse cardiovascular and cerebrovascular events. Unadjusted mode included 6 group uric acid levels. Adjusted mode included age, BMI, EF, ACS, CHF, CKD, coronary risk factors, stent type, past history, laboratory assessment (hemoglobin, platelet, HbA_1C_, eGFR and total-cholesterol) and medication *: p < 0.05. | | | | |

**Supplementary Figure**

**Figure 1S. (A)** Kaplan-Meier curve for all-cause death **(B)** Kaplan-Meier curve for MACCE.

Abbreviations: Hyper: hyperuricemic; MACCE: major adverse cardiovascular and cerebrovascular events; Normo: normouricemic.


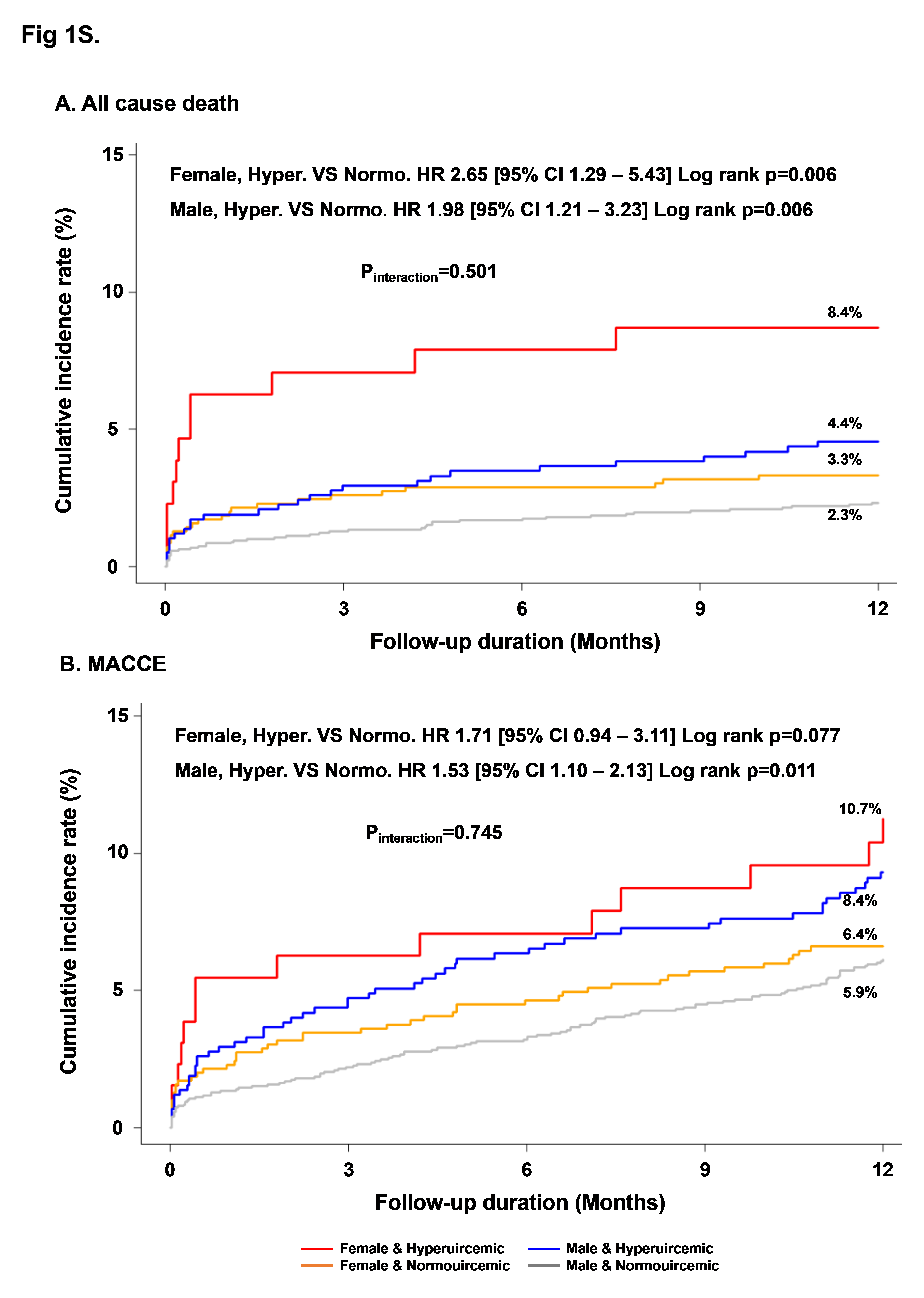


**Figure 2S. (A)** All cause death in male group. **(B)** All cause death in female group. **(C)** MACCE in male group. **(D)** MACCE in female group.

Abbreviations: ACS, acute coronary syndrome. CCS, chronic coronary syndrome. MACCE: major adverse cardiovascular and cerebrovascular events.

**
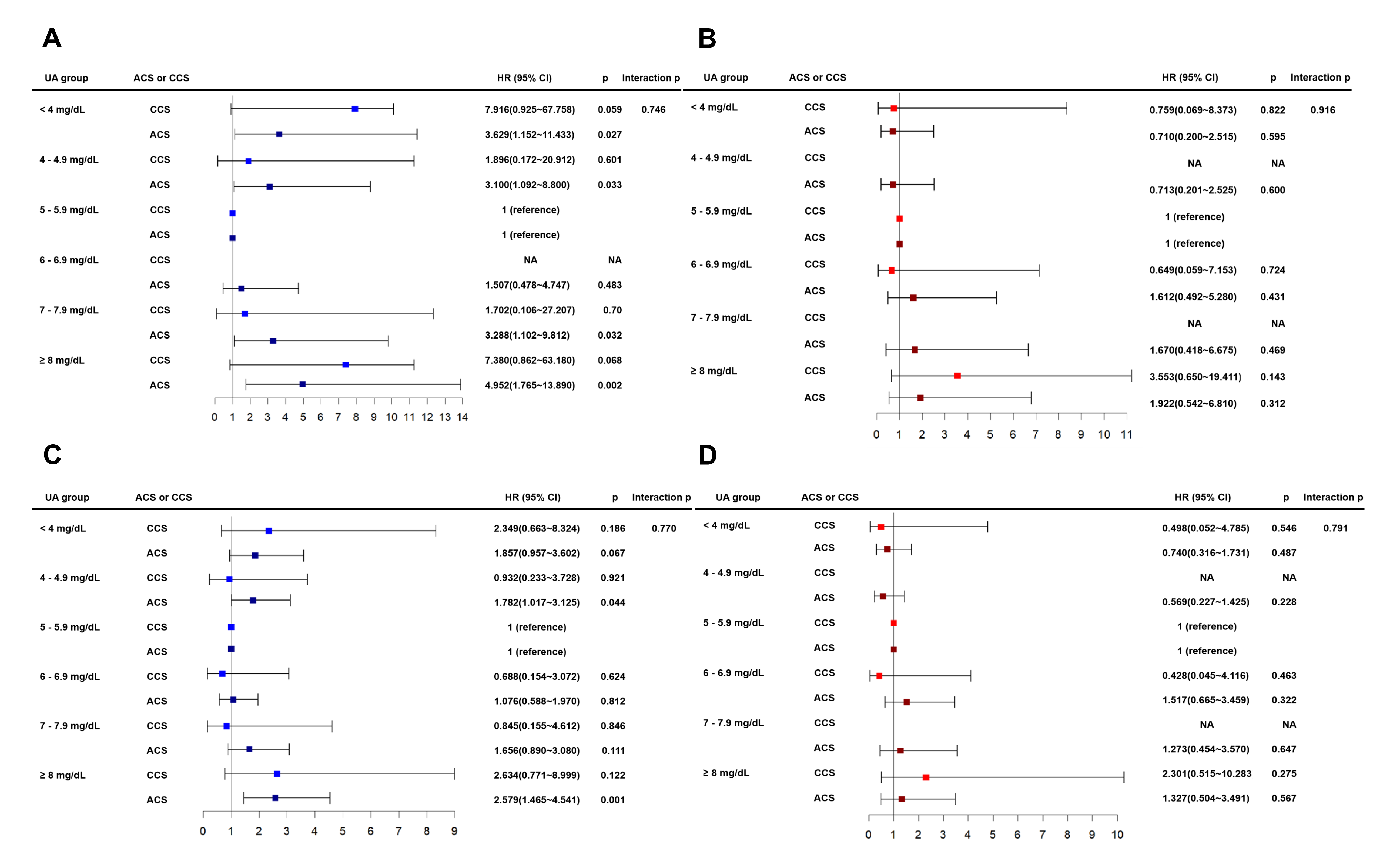
**
